# Combined metabolome and transcriptome reveal *HmF6’H1* regulating simple coumarin accumulation against powdery mildew infection in *Heracleum moellendorffii* Hance

**DOI:** 10.1186/s12870-024-05185-3

**Published:** 2024-06-06

**Authors:** Hanbing Liu, Yiran Wang, QinZheng Chang, Qiubi Li, Jiahui Fang, Ning Cao, Xuejiao Tong, Xinmei Jiang, Xihong Yu, Yao Cheng

**Affiliations:** 1 College of Horticulture and Landscape Architecture, Northeast Agricultural University, Harbin, 150030 China; 2 Key Laboratory of Biology and Genetic Improvement of Horticulture Crops (Northeast Region), Ministry of Agriculture and Rural Affairs, Northeast Agricultural University, Harbin, 150030 China

**Keywords:** *Heracleum moellendorffii* hance, *Eeysiphe heraclei*, Simple coumarins, Feruloyl CoA 6’-hydroxylase, Chemical defenses

## Abstract

**Background:**

Powdery mildew, caused by *Eeysiphe heraclei*, seriously threatens *Heracleum moellendorffii* Hance. Plant secondary metabolites are essential to many activities and are necessary for defense against biotic stress. In order to clarify the functions of these metabolites in response to the pathogen, our work concentrated on the variations in the accumulation of secondary metabolites in *H. moellendorffii* during *E. heraclei* infection.

**Results:**

Following *E. heraclei* infection, a significant upregulation of coumarin metabolites—particularly simple coumarins and associated genes was detected by RNA-seq and UPLC-MS/MS association analysis. Identifying *HmF6’H1*, a *Feruloyl CoA 6’-hydroxylase* pivotal in the biosynthesis of the coumarin basic skeleton through ortho-hydroxylation, was a significant outcome. The cytoplasmic HmF6’H1 protein was shown to be able to catalyze the ortho-hydroxylation of p-coumaroyl-CoA and caffeoyl-CoA, resulting in the formation of umbelliferone and esculetin, respectively. Over-expression of the *HmF6’H1* gene resulted in increased levels of simple coumarins, inhibiting the biosynthesis of furanocoumarins and pyranocoumarins by suppressing PT gene expression, enhancing *H. moellendorffii* resistance to powdery mildew.

**Conclusions:**

These results established *HmF6’H1* as a resistance gene aiding *H. moellendorffii* in combatting *E. heraclei* infection, offering additional evidence of feruloyl-CoA 6’-hydroxylase role in catalyzing various types of simple coumarins. Therefore, this work contributes to our understanding of the function of simple coumarins in plants’ defense against powdery mildew infection.

**Supplementary Information:**

The online version contains supplementary material available at 10.1186/s12870-024-05185-3.

## Background

*Heracleum moellendorffii* Hance, a member of the Umbelliferae order, is well-known for both its flavor and its abundance of active ingredients, including coumarins, which have been shown to have remarkable anti-cancer properties [1]. As an understory herb, wild *H. moellendorffii* cannot meet market demands, necessitating the artificial cultivation of this plant in fields and greenhouses. However, the powdery mildew caused by Eeysiphe heraclei poses a significant threat to all developmental stages of *H. moellendorffii*, affecting seeds, flowers, and leaves. This results in compromised plant quality, leading to yield losses ranging from 10 to 40% and impeding large-scale cultivation [2]. Nevertheless, no genes resistant to *E. heraclei* have been found in *H. moellendorffii*. Currently, chemical control has little effect on the leaf characteristics and phytotoxicity of *H. moellendorffii*, which develops in facilities. While, the osthol-based botanical fungicides were reported to have efficient powdery mildew-controlling properties [3]. Therefore, coumarin-based botanical fungicides derived from natural products offer an alternative for protecting plants that could reduce risks and their effects on the environment and public health [4].

Coumarins, plant secondary metabolites, are synthesized in monocotyledonous and dicotyledonous plant species, maintaining exceptionally high levels in the Umbelliferae, Rutaceae, and Compositae species. The initial step in coumarin synthesis involves the ortho-hydroxylation of cinnamates, branching from the lignin biosynthesis derived from the phenylpropane metabolic pathway [5]. The “basic coumarin,” or structural core of coumarins, is 2 H-1-benzopyran-2-one. Modifications to this core allow coumarins to be categorized into two groups: complex and simple [6]. Simple coumarins, including scopolin, scopoletin, esculin, esculetin, and umbelliferone, serve as naturally occurring phytoalexins involved in crucial biological processes, particularly as signaling molecules controlling the growth and multiplication of various plant pathogens [6–9]. For example, scopoletin is suggested to prevent or reduce diseases caused by necrotrophic fungi *Alternaria alternata* and *Pseudomonas syringae* pv. *tabaci* in tobacco and the bacterial pathogen *Pseudomonas syringae* in *Arabidopsis* [10–13]. Scopolin, a glycoside form of scopoletin, likely contributes to plant pathogen resistance [14]. In addition to being substrates for the synthesis of furanocoumarins, pyranocoumarins, and other simple coumarins, esculin and 7-hydroxycoumarin umbelliferone are involved in defense against *Penicillium digitatum* and several other pathogenic fungi [15–17]. Although coumarin metabolites control powdery mildew, only osthol-based botanical fungicides have been shown to have efficient powdery mildew-controlling properties [3]. Therefore, it is urgent to develop several new botanical fungicides to control powdery mildew occurring in *H. moellendorffii* in facilities. *H. moellendorffii* contains the active substance coumarins, and therefore, coumarin product-based botanical fungicides would be an appealing strategy for effective agronomic application against powdery mildew caused by *H. moellendorffii* in facilities.

In plants, the production of basic coumarins includes the process of ortho-hydroxylation of corresponding cinnamates. This occurs at the position adjacent to the side chain on the benzene ring [5, 13]. This process is mediated by the 2-oxoglutarate-dependent dioxygenase (2OGD), designated explicitly as feruloyl CoA 6’-hydroxylase (F6’H), which is recognized as a key gene in the biosynthesis of simple coumarins via ortho-hydroxylation [18, 19]. Previous research has explored the ortho-hydroxylation function of F6’H in various plants. *AtF6’H1* and *AtF6’H2* in *Arabidopsis* demonstrate ortho-hydroxylation activity on feruloyl-CoA, resulting in the formation of scopoletin. However, they do not show any effect on p-coumaroyl-CoA and caffeoyl-CoA 3. *IbF6’H2* in sweet potato (*Ipomoea batatas*) catalyzes the ortho-hydroxylation of p-coumaroyl-CoA and feruloyl CoA, resulting in the production of umbelliferone and scopoletin, respectively. However, IbF6’H1 solely catalyzes ortho-hydroxylation to feruloyl-CoA [20]. These studies reveal the complex substrate specificity of the ortho-hydroxylation function of F6’H. Moreover, *F6’H* genes have been implicated in playing a vital role in protecting against fungal diseases by controlling the accumulation of basic coumarins at the sites of infection by the pathogen [10, 13, 21, 22]. The F6’H genes regulating simple coumarins in plant disease resistance may require signaling molecule activation [6]. Previous studies have demonstrated that hormones such as jasmonic acid (JA) and ethylene (ET) stimulate the development of Na *F6’H1*, which leads to the accumulation of simple coumarin at the site of infection by *A. alternata* in tobacco leaves [13, 14, 23]. In addition, the oxidative burst can induce AtF6’H1 expression and regulate coumarin accumulation, defending the pathogen’s attack [24]. However, there is a considerable lack of evidence concerning the potential response of simple coumarins and *F6’H* genes to powdery mildew infection. This impedes our ability to comprehend the precise function of coumarin metabolites in powdery mildew resistance in plants, which is detrimental to the ensuing development of powdery mildew control for *H. moellendorffii*. To breed powdery mildew resistance in *H. moellendorffii* and develop new plant-derived botanical fungicides, it is crucial to investigate the accumulation and distribution of coumarin comparable under powdery mildew infestation, uncover the critical genes involved in this process and examine the role of coumarin analogs in the resistance mechanism of plants against powdery mildew.

Coumarins can absorb UV light, producing distinctive fluorescence [25]. Previous studies have shown that the infective pathogen sites emit strong fluorescence under UV light due to the accumulation of simple coumarins around these areas [13]. The present study observed intense fluorescence in places affected by powdery mildew. Based on this observation, it was hypothesized that there might be a specific accumulation of coumarins in the leaves following powdery mildew infection. Therefore, the present study analyzed the transcriptomic and metabolomic dynamics during different periods of *E. heraclei* infection. It was found that coumarin metabolites were involved in the plant’s response to powdery mildew infection. Hence, this study analyzed coumarin accumulation patterns following powdery mildew infection using UPLC-MS/MS technology. The RNA sequencing (RNA-seq) platform identified the key feruloyl CoA 6’-hydroxylase gene, *HmF6’H1*, which exhibited ortho-hydroxylation activity. Prokaryotic expression in *E. coli* BL21 (DE3) confirmed the ortho-hydroxylation activity of *HmF6’H1*. Furthermore, transient expression demonstrated that *HmF6’H1* was a resistant gene that resisted *E. heraclei* infection. The *HmF6’H1* gene cloned in this study will be a valuable resource for future studies of powdery mildew resistance and coumarin-rich crop breeding programs. In the meanwhile, simple coumarins offers a desirable natural ingredient for the development of botanical fungicides and directs the production of green prevention and control of powdery mildew in coumarin-rich plants.

## Results

### *Heracleum moellendorffii* metabolome and transcriptome were co-regulated and corresponded to the developmental stages of *E. heraclei* infection

**Fig. 1:**
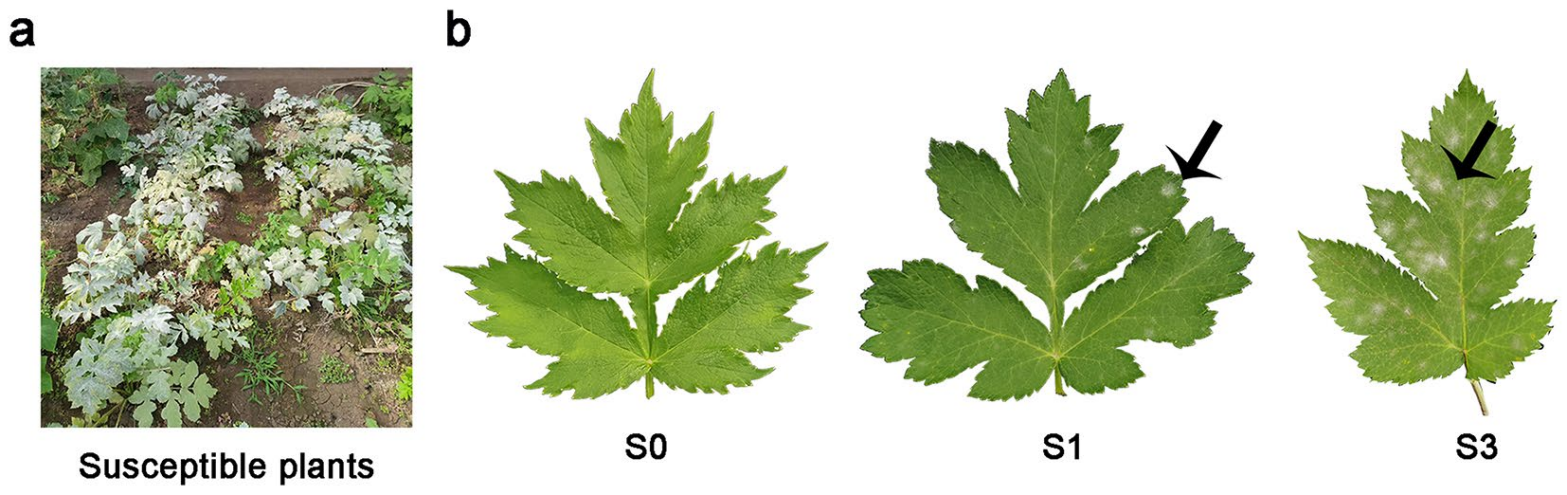
The phenotypic characteristics of *Heracleum moellendorffii* following *E. heraclei* infection. (**a**) Three-year-old *H. moellendorffii* is a highly susceptible plant. (**b**) Leaf samples were used for RNA-seq and UPLC-MS/MS analysis

In order to more fully understand the changes in primary and secondary metabolites caused by *E. heraclei* infection, the leaves at various stages (S0, S1, and S3) were systematically collected, as shown in Fig. 1a and b.

**Fig. 2:**
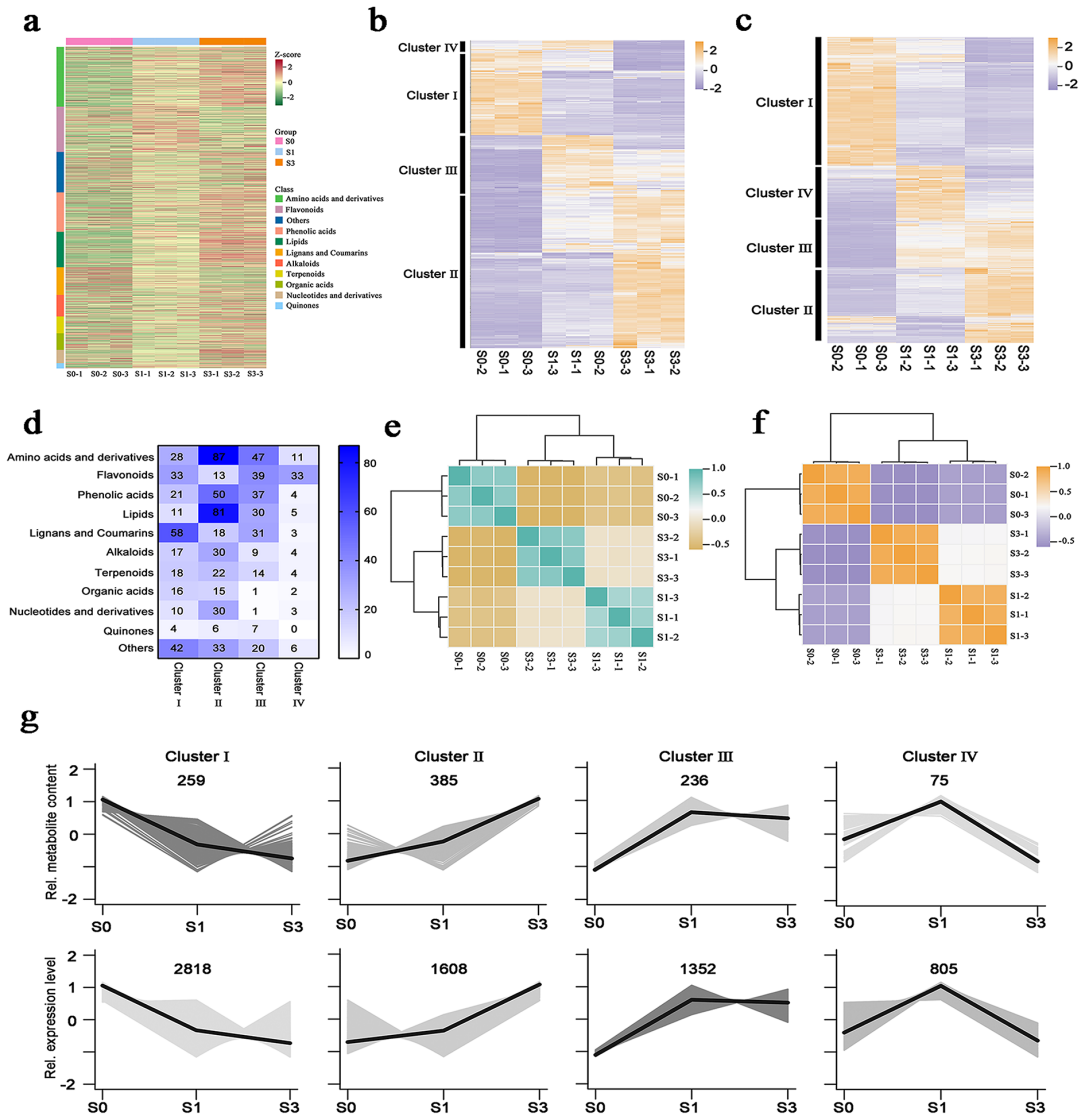
Summary of *H. moellendorffii* transcriptomic and metabolomic data. (**a**) Overview of the 1,700 annotated metabolites. (**b-c**) Expression patterns of 955 DAMs (**b**) and 6,602 DEGs (**c**) in leaves at different infected periods. (**d**) The accumulation patterns of 955 DAMs in Clusters I, II, III;, and IV. (**e-f**) The hierarchical clustering analysis of the expression profiles of 1,700 metabolites (**e**) and 19,245 genes (**f**). (**g**) The K-means clustering algorithm categorized the expression profiles of the *H. moellendorffii* transcriptome and metabolome into four distinct clusters in leaves at various stages of infection

A comprehensive analysis using the UPLC-MS/MS platform identified a total of 1,700 metabolites during the three infection periods. These encompassed 316 amino acids and derivatives, 239 flavonoids, 209 phenolic acids, 187 lipids, 146 lignans and coumarins, 114 alkaloids, and others (Fig. 2a, Table S1). A total of 955 differentially accumulated metabolites (DAMs) across various infection stages were identified (Table S2) and categorized into four large groups through clustering analysis and K-means (Fig. 2b and g, Table S3). Each cluster exhibited distinct metabolic patterns consistent with the respective infection period. Notably, the accumulation of metabolites differed over the three infection phases. Clusters I and III mainly acquired a higher concentration of metabolites such as coumarins, flavonoids, amino acids, and derivatives. In comparison, Cluster II showed an accumulation of other metabolites, such as amino acids and derivatives, lipids, and phenolic acids (Fig. 2d, Table S3). The observed metabolite patterns likely indicated the variation in *H. moellendorffii* metabolism as it responded to the development of powdery mildew infection.

To examine the control of transcription after powdery mildew infection, RNA-seq analysis was utilized, resulting in the identification of 6,602 differentially expressed genes (DEGs) in all samples (Table S4). These DEGs were categorized into four major groups based on the expression patterns, demonstrating consistent and distinct expression patterns aligned with the accumulation of DAMs (Fig. 2c and g). Cluster II, comprising 1,608 genes, exhibited increasing expression levels from the S0 to the S1 and S3 periods, indicating a positive association with the progression of powdery mildew infection. In particular, these genes were found to have an abundance of protein kinase domains and cytochrome P450 genes, indicating a crucial function in transferring signals through protein phosphorylation and modifying secondary metabolites. Moreover, Cluster II enriched several transcription factors, including bHLH and WRKY, implying their critical roles in powdery mildew infection.

A consistent expression pattern between DEGs and DAMs was shown in the correlation matrix, demonstrating the accumulation of specific metabolites after powdery mildew infection (Fig. 2e and f). These results collectively suggest a pronounced specificity in gene expression and metabolite accumulation throughout the three infection stages of powdery mildew.

**Fig. 3:**
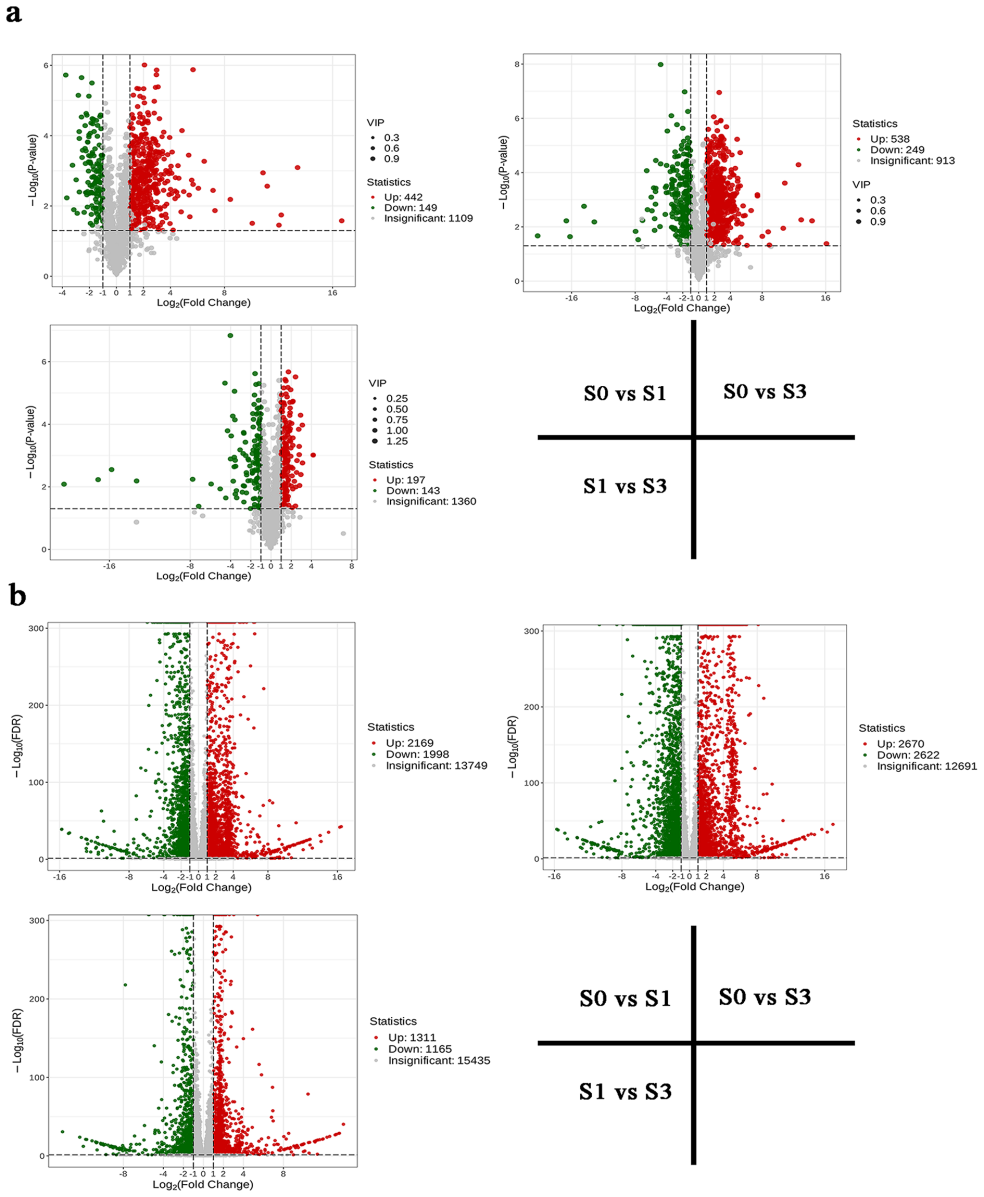
DAMs (**a**) and DEGs (**b**) in *H. moellendorffii* leaves at different infected periods (VIP > 1, fold change ≥ 2, or fold change ≤ 0.5)

### Identification of the DAMs and DEGs following *E. heraclei* infection

**Fig. 4:**
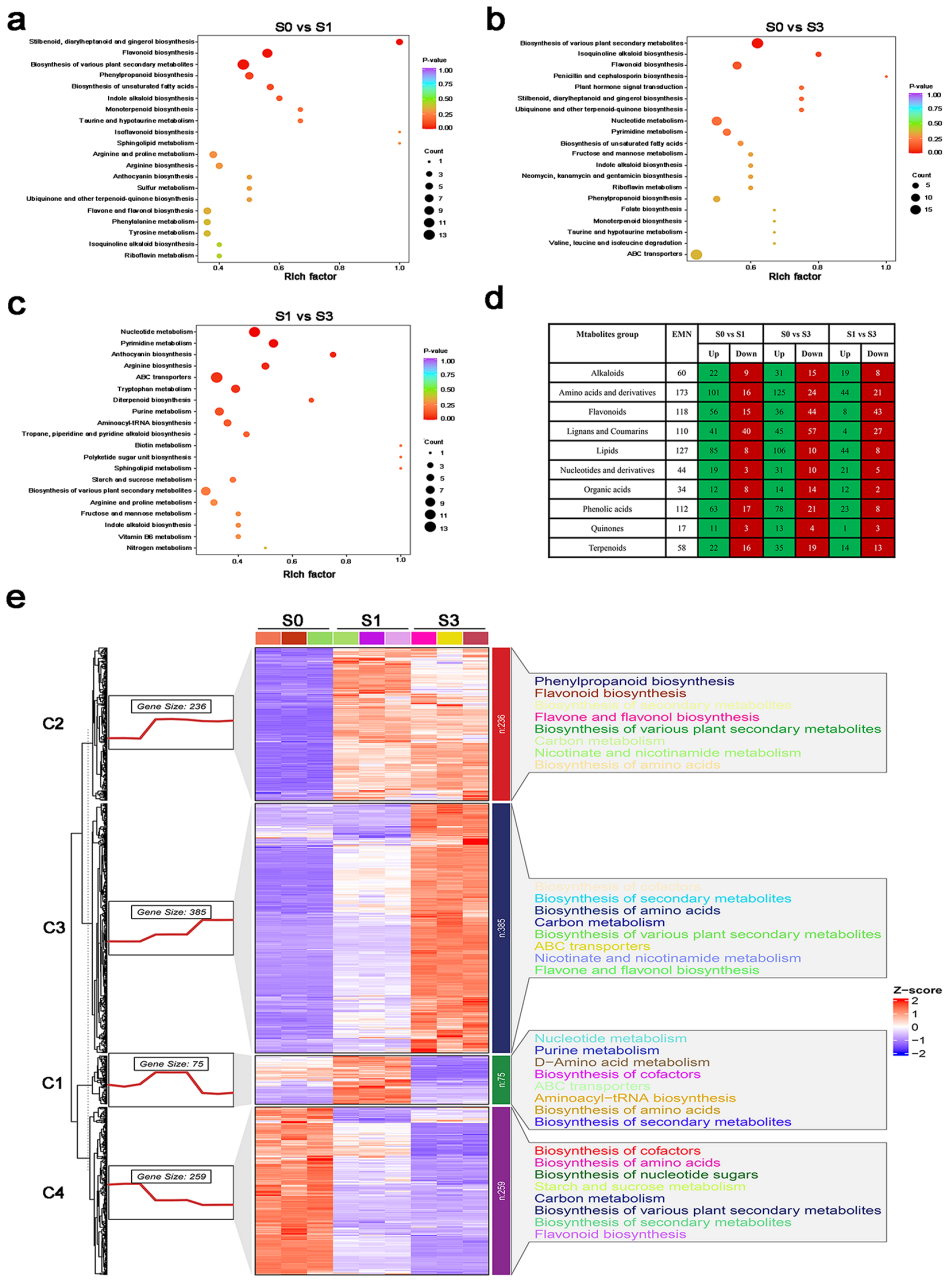
Identification and functional characterization of DAMs in *H. moellendorffii* leaves at different infected periods. (a-c) KEGG enrichment analysis of the DAMs between (**a**) S0 vs. S1, (**b**) S0 vs. S3, and (**c**) S1 vs. S3. (**d**) Functional categorization of DAMs in three groups was compared based on metabolite groups. (**e**) KEGG enrichment of four metabolite clusters is based on similarities in their expression pattern

DAMs between *H. moellendorffii* samples (S0 vs. S1, S0 vs. S3, S1vs S3) were identified based on the variable importance in projection (VIP > 1, fold change ≥ 2, or fold change ≤ 0.5) (Table S2). Further analysis revealed a significant accumulation of DAMs between the compared samples, with 591, 787, and 340 DAMs in S0 vs. S1, S0 vs. S3, and S1 vs. S3, respectively (Fig. 3). DAMs found in all examined samples were considerably abundant in flavonoid biosynthesis, nucleotide metabolism, phenylpropanoid biosynthesis, and the production of other plant secondary metabolites, according to KEGG enrichment analysis (Fig. 4a-c). To gain further understanding of how the 955 DAMs responded to E. *heraclei* infection, Mfuzz categorized these DAMs into four clusters based on their expression pattern similarities (Fig. 4e). Following *E. heraclei* infection, the metabolites in Clusters C2 and C3 were upregulated and demonstrated significant enrichment in phenylpropanoid biosynthesis, secondary metabolite biosynthesis, various plant secondary metabolite biosynthesis, carbon metabolism, and flavonoid biosynthesis, among other metabolites. These upregulated metabolites focused on amino acids and derivatives, lignans and coumarins, flavonoids, and phenolic acids (Fig. 4d). The results suggested that these metabolites may be crucial in resisting *E. heraclei* infection.

**Fig. 5:**
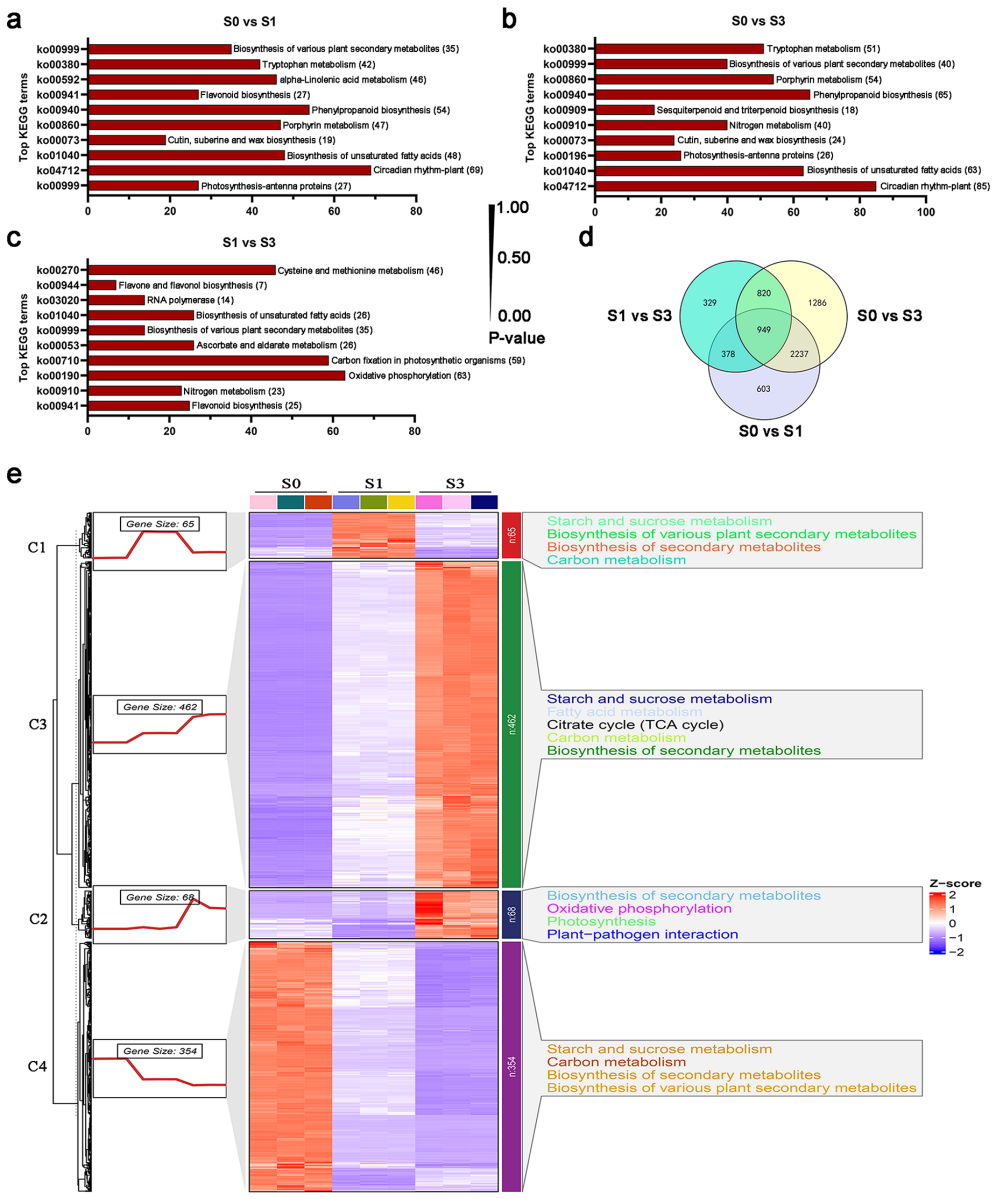
Identification and functional characterization of DEGs in *H. moellendorffii* leaves at different infected periods. (a-c) KEGG enrichment analysis of the DEGs between (**a**) S0 vs. S1, (**b**) S0 vs. S3, and (**c**) S1 vs. S3. (**d**) Venn diagram between S0 vs. S1, S0 vs. S3, and S1 vs. S3. (**e**) KEGG enrichment of four gene clusters is based on similarities in their expression pattern

An analysis of DEGs between the compared samples was conducted to validate the dynamic nature of DEG changes throughout the three infected periods of powdery mildew. A total of 4,167, 5,292, and 2,476 DEGs were identified for S0 vs. S1, S0 vs. S3, and S1 vs. S3, respectively (Fig. 3). The top enriched KEGG terms contributed by these DEGs were primarily distributed in ko00196 (photosynthesis-antenna proteins), ko01040 (biosynthesis of unsaturated fatty acids), ko00940 (phenylpropanoid biosynthesis), ko00941 (flavonoid biosynthesis), ko00999 (biosynthesis of various plant secondary metabolites), and ko00910 (nitrogen metabolism) (Fig. 5a-c). All DEG samples were clustered to identify the conserved genes consistently expressed in S0, S1, and S3 infected periods (Fig. 5d, Table S5). This study yielded 949 DEGs when comparing three groups. This indicates that these genes, which are conserved and essential, may play a role in the interaction between powdery mildew and *H. moellendorffii*. To provide a better comprehension of how the 949 DEGs reacted to *E. heraclei* infection, Mfuzz categorized these DEGs into four clusters according to their similarities in expression patterns (Fig. 5e). These conserved DEGs were predominantly enriched in phenylpropanoid biosynthesis, starch, and sucrose metabolism, biosynthesis of secondary metabolites, biosynthesis of various plant secondary metabolites, and carbon metabolism. Furthermore, MYB and C2C2-CO-like transcription factors were enriched among conserved DEGs (Table S5).

### Coumarin metabolites were involved in *E. heraclei* infection in *H. moellendorffii*

**Fig. 6:**
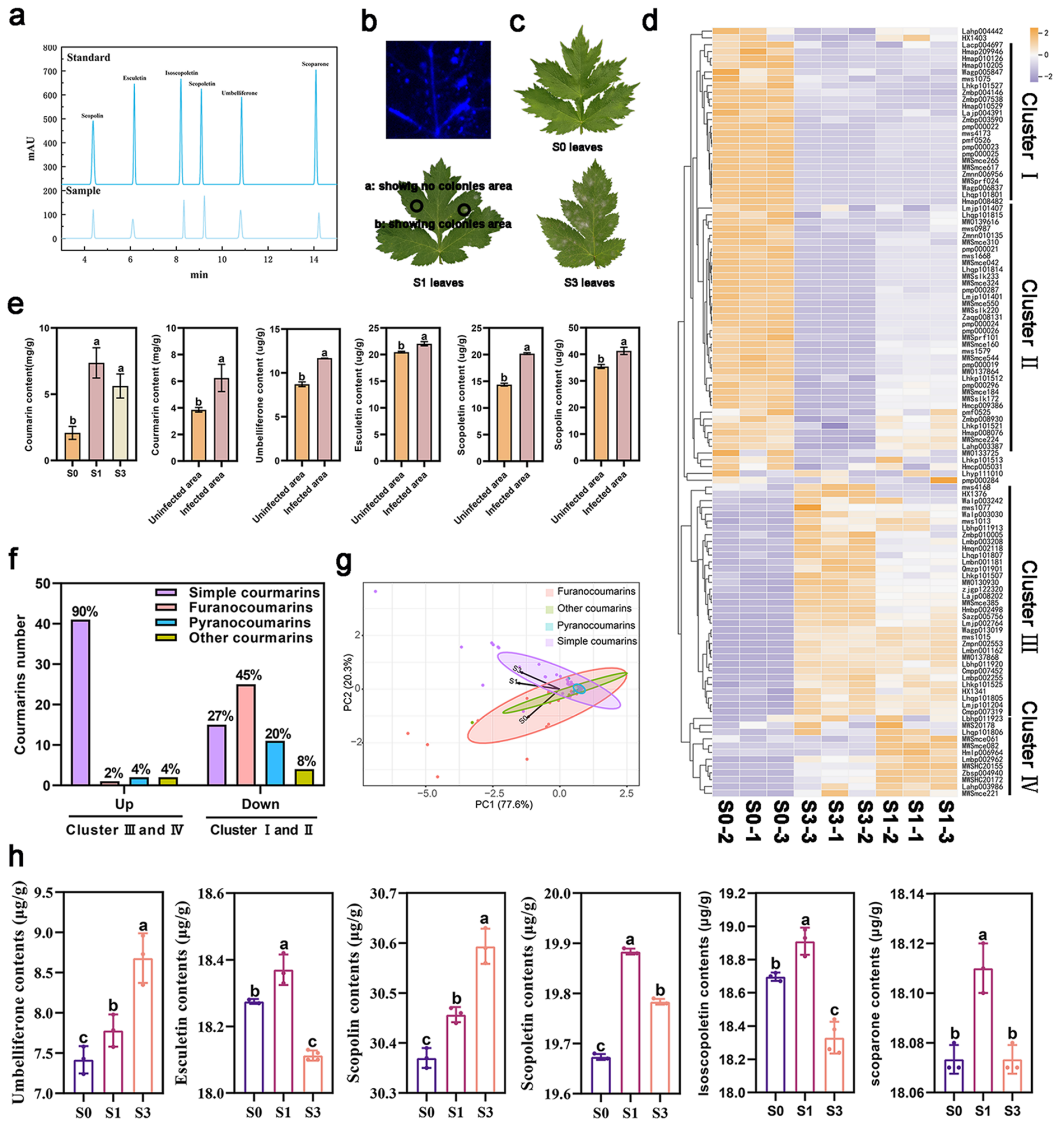
HPLC and UPLC-MS/MS coumarin metabolite analysis in *H. moellendorffii* after *E. heraclei* infection. (**a**) The peak HPLC plot of standards and samples. (**b**) Strong blue fluorescence was present around the inoculation sites. (**c**) Leaf sample schematic for HPLC analysis of coumarin metabolites. a: showing no colonies in S1 leaves. b: showing colonies in S1 leaves. (**d**) Expression patterns of 113 coumarin metabolites in leaves at three infective periods. (**e**) The content of simple coumarin in leaves following *E. heraclei* infection. (**f**) The percentage of different coumarin types of the up and down-regulated coumarin metabolites in S1 and S3 leaves. (**g**) PCA analysis of coumarin metabolites in leaves at three infective periods. (**h**) The content of simple coumarins of S0, S1, and S3 infected leaves

The KEGG analysis showed that these accumulated secondary metabolites were mainly focused on coumarin metabolites (Fig. 4d). In this study, the presence of strong fluorescence in the powdery mildew infective areas suggested a specific accumulation of coumarins in leaves after powdery mildew infection (Fig. 6b). Moreover, further analysis mainly focused on exploring the accumulation change of coumarins after *E. heraclei* infection. The results showed infected leaves at stages S1 and S3 had significantly higher total coumarin levels than uninfected leaves at stage S0.

Furthermore, the infected leaves showed higher levels of total coumarin, umbelliferone, esculetin, scopoletin, and scopolin in the visible colony sites compared to the uncolonized sites (Fig. 6a, c, and e). The findings indicated that the levels of coumarins, particularly simple coumarins, were briefly modified after *E. heraclei* infection. A total of 113 monomers with coumarin-like properties were found, comprising 53 basic coumarins, 26 furanocoumarins, 17 pyranocoumarins, and 8 different types of coumarins (Table S6). The accumulation patterns of these metabolites in S0, S1, and S3 leaves were analyzed (Fig. 6d). Compared with S0 leaves, coumarin-like monomers in Clusters III and IV were upregulated in S1 and S3 leaves. S1 and S3 leaves showed significant enrichment in particular simple coumarins, such as umbelliferone, esculetin, scopoletin, and scopolin, whereas other coumarins demonstrated lower enrichment levels. In Clusters I and II, the levels of metabolites in S1 and S3 leaves were reduced. There was a higher level of furanocoumarins and pyranocoumarins, but the proportion of simple coumarin metabolites decreased (Fig. 6d and f). PCA analysis further demonstrated that simple coumarins exhibited the largest difference within the 113 coumarin metabolites in S0, S1, and S3 leaves, followed by the furanocoumarins (Fig. 6g). The findings indicated that the accumulation of simple coumarins occurred at various stages during the infection process of *E. heraclei*. These coumarins may interact with *H. moellendorffii* and powdery mildew and play crucial roles in reacting to *E. heraclei* infection. The HPLC study confirmed a considerable increase in the concentration of umbelliferone, esculetin, scopoletin, scopolin, scopoletin, and scoparone in S1 or S3 leaves compared to S0 leaves, which is consistent with the findings of the UPLC-MS/MS analysis (Fig. 6h).

### Identification of crucial candidate genes involved in simple coumarin metabolism following *E. heraclei* infection

**Fig. 7:**
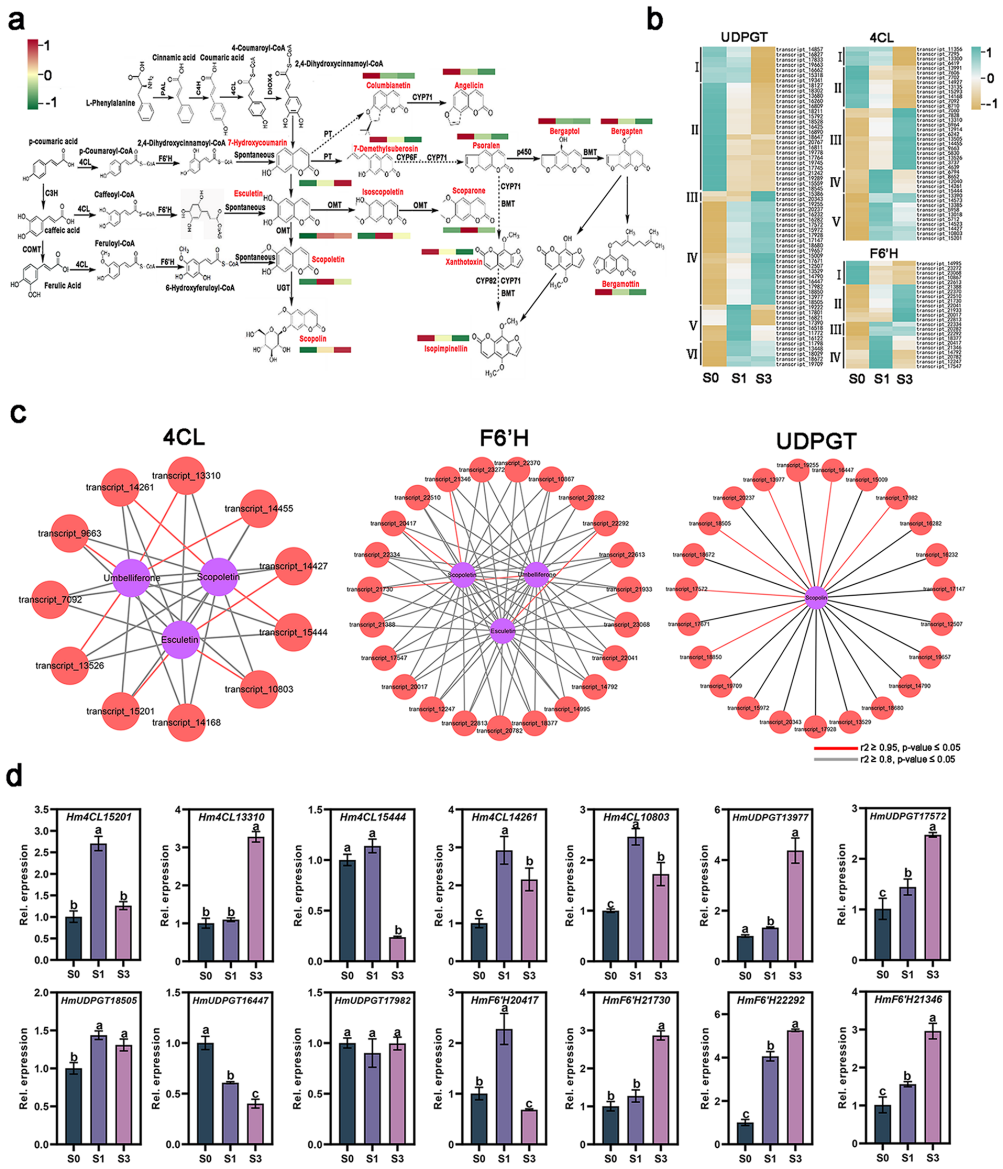
Gene expression analysis of S0, S1, and S3 infected leaves. (**a and b**) The expression of genes involved in coumarin metabolite biosynthesis. (**c**) A network assembled from correlations among structural genes and simple coumarins (r^2^ > 0.95, *p* < 0.01). The red lines represent r^2^ > 0.95, *p* < 0.05, and the black lines represent r^2^ > 0.8, *p* < 0.05. (**d**) The expression levels of *Hm4CL*, *HmUDPGT*, and *HmF6’H* genes in S0, S1, and S3 infected leaves

According to the analysis provided, simple coumarins may be involved in the interaction between *H. moellendorffii* and powdery mildew, as shown in Fig. 6d-g. Umbelliferone and esculetin are the fundamental structures of simple coumarins, which serve as the foundation for developing furanocoumarins, pyranocoumarins, and other comparable substances [26]. In the catalytic process, simple coumarins were all upregulated. In contrast, furanocoumarins and pyranocoumarins were all down-regulated in S1 and S3 infected leaves, compared to S0 leaves (Fig. 7a). This study primarily aimed to investigate the specific functions of simple coumarins in their response to powdery mildew infection. Coumarin, a compound derived from hydroxycinnamic acid, is synthesized by the action of two enzymes: 4-cinnamic acid: coenzyme A ligase (4CL) and *F6’H*. Scopolin, a ß-glycoside form of scopoletin, is possibly catalyzed by *scopoletin glucosyltransferase* (*UDPGT*) (Fig. 7a). The expression patterns of *4CL, F6’H*, and *UDPGT* genes were analyzed (Fig. 7b). *UDPGT* genes were divided into six clusters based on gene expression levels. Genes in Cluster IV; (such as transcript_ 13,448, 11,798, and 18,029) were significantly upregulated in S1 and S3 infected leaves. In contrast, the expression of Cluster II genes, including transcript_18211, 15,792, and 18,528, was significantly suppressed compared to S0 leaves. The expression patterns of *4CL* genes were categorized into five clusters. Genes in ClusterV (such as transcript_15201, 10,803, and 14,427) were induced obviously in S1 and S3 leaves, while genes in Cluster II (such as transcript_13991, 7606, and 7702) were down-regulated compared with S0 leaves. *F6’H* genes were divided into four clusters based on expression patterns. In S1 and S3 infected leaves, genes in Cluster III (such as transcript_ 22,292, 20,282, and 22,334) were upregulated. The expression levels of genes in Cluster I (14,995, 23,068, and 10,867) were inhibited compared with S0 leaves.

A network has been established to regulate the co-expression between necessary enzymes and metabolites to discover prospective genes associated with the biosynthesis of simple coumarin. Pearson correlation analysis (r^2^ ≥ 0.95, p-value < 0.05) demonstrated a positive correlation between *4CL* (transcript_13310, 14,261, 9663, 13,526, 15,201, 10,803, 15,444, 14,427, and 14,455) and *F6’H* genes (transcript_22292, 21,346, 20,417, and 21,730) with the accumulation of umbelliferone, esculetin, and scopoletin. Additionally, *UDPGT* genes (transcript_17982, 16,447, 13,977, 18,505, 17,572, and 18,850) showed a strong correlation with the accumulation of scopolin. These results indicated that these catalases may have a role in the biosynthesis of simple coumarin (Fig. 7 c, Table S7). qRT–PCR analysis of these genes produced results that were highly consistent with the RNA-seq data (Fig. 7d). Moreover, *4CL* (transcript_14261 and 10,803), *UDPGT* (transcript_17572 and 18,505), and *F6’H* genes (transcript_20417, 22,292, and 21,346) were significantly upregulated in S1 and S3 leaves, indicating their potential involvement in *E. heraclei* infection.

### Functional analysis of the HmF6’H1 protein

**Fig. 8:**
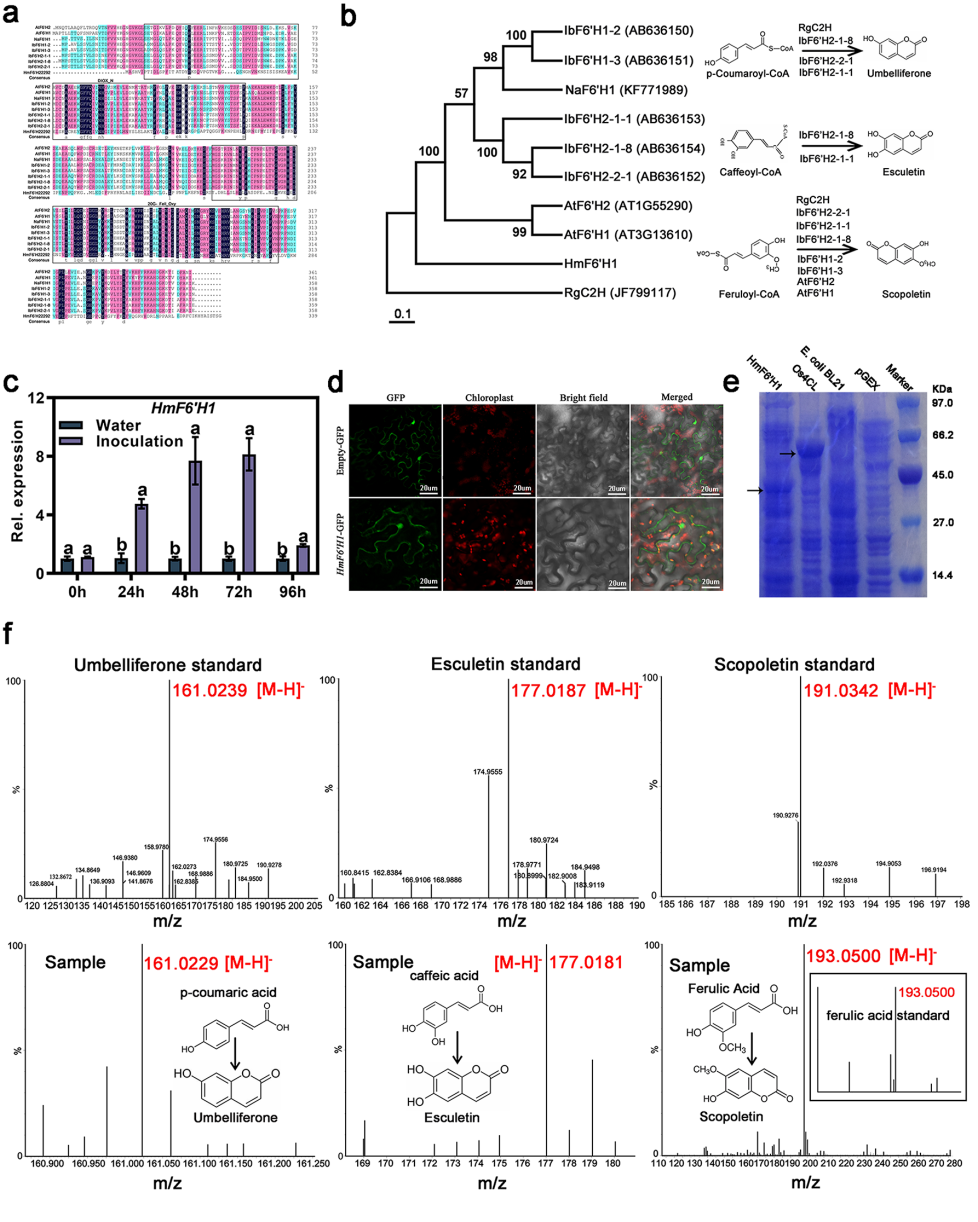
The functional verification of HmF6’H1 proteins in *H. moellendorffii*. (**a**) Multiple sequence alignment of *HmF6’H1* with *AtF6’H*, *NaF6’H*, and *IbF6’H* genes. (**b**) A phylogenetic neighbor-joining tree of HmF6’H1 in *H. moellendorffii* with AtF6’H, NaF6’H, IbF6’H, and RgC2H. (**c**) HmF6’H1 expression following artificial inoculation. (**d**) Subcellular localization of HmF6’H1-GFP transiently expressed in *N. benthamiana* epidermal cells. Scale bar: 20 μm. (**e**) Coomassie-stained SDS-PAGE gel of Os4CL and HmF6’H1 expressed in *E. coli* BL21. (f) UPLC-QTOF-MS analysis of enzymatic reaction products of *E. coli* BL21 expressing pGEX-HmF6’H1 and pGEX-Os4CL

The *F6’H* is a crucial gene in biosynthesis, whereby ortho-hydroxylation forms the fundamental framework of coumarin [5]. This study identified *HmF6’H1* (transcript_22292) and *HmF6’H2* (transcript_20417) genes among the 949 conserved DEGs were upregulated in three compared with groups (Fig. 5d and e, Table S5). This led to the assumption that *HmF6’H1* and *HmF6’H2* may be involved in the interaction between *E. heraclei* and *H. moellendorffii*. Only the *HmF6’H1* gene, however, was effectively cloned; the substantial genomic heterozygosity of the *HmF6’H2* gene made sequence splicing accuracy difficult. The results of multiple sequence alignment showed that the HmF6’H1 protein contains various conserved amino acid residues, such as proline (pro or p), glutamine (Gln or q), and glycine (Gly or g), in combination with conserved structural motifs including DIOX_N and 2OG-Fell_OXY (Fig. 8a). Phylogenetic analysis demonstrated that HmF6’H1 did not cluster with F6’H proteins from other plants but formed a distinct branch (Fig. 8b). *HmF6’H1* responded to *E. heraclei* infection, showing a noticeable up-regulation in expression levels at 24, 48, 72, and 96 h with artificial inoculation compared to water treatment (Fig. 8c). HmF6’H1 was primarily localized to the cytoplasm, according to subcellular localization studies (Fig. 8d). In BL21 (DE3) cells, pGEX-HmF6’H1, and pGEX-Os4CL were produced to examine the enzymatic activity of HmF6’H1 proteins (Fig. 8e). P-coumaric acid, caffeic acid, and ferulic acid were the substrates used to assay the IPTG-induced cultures. Umbelliferone and esculetin, the expected results of ortho-hydroxylation of p-coumaroyl-CoA and caffeoyl-CoA, were produced by HmF6’H1 proteins. However, HmF6’H1 proteins did not catalyze the ortho-hydroxylation of feruloyl-CoA(Fig. 8f). 

### *HmF6’H1* positively regulated *H. moellendorffii* resistance to *E. Heraclei*

**Fig. 9:**
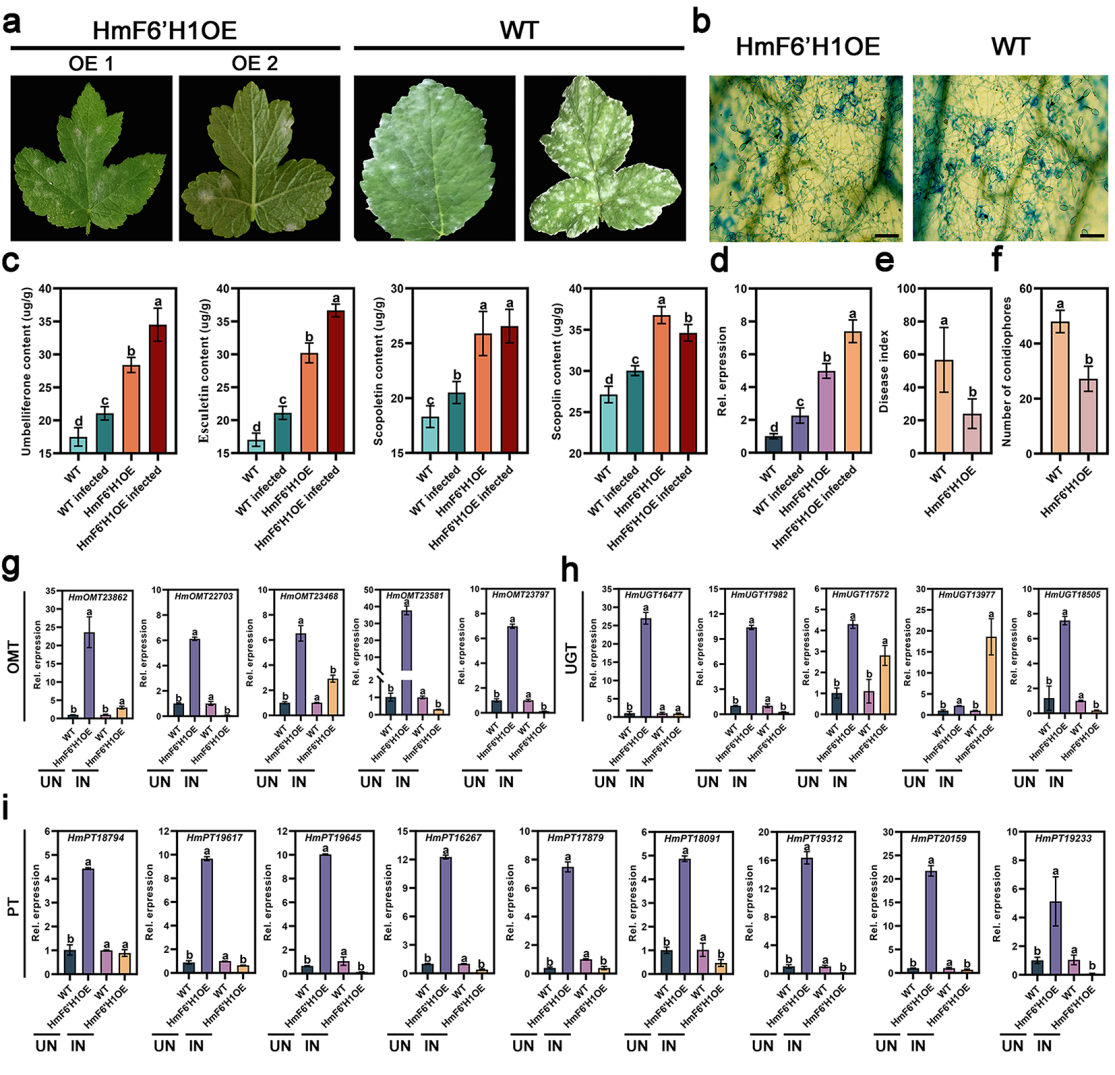
Transient expression of HmF6’H1 in *H. moellendorffii*. (**a**) Leaves of typical *H. moellendorffii* genotypes infected with *E. heraclei* at 10 dpi. (**b**) Representative images of *H. moellendorffii* leaves of various genotypes infected with *E. heraclei* at 10 d. Scale bar: 20 μm. (**c**) The content of simple coumarins in WT, infected WT, HmF6’H1OE, and infected HmF6’H1OE plants. (**d**) The expression levels of HmF6’H1 in WT, infected WT, HmF6’H1OE, and infected HmF6’H1OE plants. (e-f) The leaf disease index (**e**) and conidiophores per colony (**f**) in WT and HmF6’H1OE plants. (**g-i**) The expression levels of OMT (**g**), UGT (**h**), and PT (**i**) in WT and HmF6’H1OE plants without (UN) and with (IN) *E. heraclei* infection

*H. moellendorffii* was used in a transient overexpression experiment to examine the function of *HmF6’H1* during *E. heraclei* infection. Figure 9 d shows that *HmF6’H1* expression was substantially upregulated in overexpression plants (OE) as opposed to the wild-type (WT), suggesting that *H. moellendorffii* leaves were successfully overexpressed. Following *E. heraclei* infection, *HmF6’H1* was significantly upregulated in infected HmF6’H1OE plants compared to both HmF6’H1OE and infected WT plants (Fig. 9d). Phenotypic analysis demonstrated that disease severity was lower in HmF6’H1OE plants, with a lower disease index than WT plants after *E. heraclei* infection at 10 days post-infection (10dpi) (Fig. 9a and e). Pathological observation confirmed this conclusion, as more conidiophores germinated in WT plants than in HmF6’H1OE plants (Fig. 9b and f). The results suggested that HmF6’H1 could be considered a resistance gene, enhancing *H. moellendorffii’s* resistance to *E. heraclei*. Umbelliferone, esculetin, scopoletin, and scopolin content were measured to validate further simple coumarins’ involvement in responding to *E. heraclei* infection. The findings demonstrated that the four simple coumarins in uninfected and infected HmF6’H1OE plants were substantially higher than in infected WT plants. (Figure. 9c). The level of scopoletin and scopolin in both infected and uninfected HmF6’H1OE plants was much higher than that of infected WT, even though HmF6’H1 did not demonstrate enzymatic activity towards feruloyl-CoA (Fig. 9f). Therefore, the expression of essential genes such as O-methyltransferase (OMT) and glucosyltransferases (UGT) in the catalytic pathway from umbelliferone and esculetin to scopoletin and scopoline may be affected by the expression of HmF6’H1. qRT-PCR analysis revealed that when HmF6’H1 was overexpressed, the expression levels of *OMT* genes (except *HmOMT23468*) following *E. heraclei* infections were significantly inhibited compared to plants without *E. heraclei* infection (Fig. 9g). The *UGT* genes (except *HmUGT17572* and *HmUGT13977*) exhibited a consistent trend with *OMT* genes (Fig. 9h). Moreover, prenyltransferase (PT), an essential rate-limiting enzyme catalyzing umbelliferone formation of furanocoumarins and pyranocoumarins by adding a prenyl group [27], was significantly down-regulated in HmF6’H1OE following *E. heraclei* infection compared to uninfected plants (Fig. 9i). Therefore, the down-regulation of PT genes brought about by the *E. heraclei* infection further impeded the production of furanocoumarins and pyranocoumarins (Fig. 7a). These findings showed that *HmF6’H1* overexpression might affect the accumulation and distribution of simple coumarins, furanocoumarins, and pyranocoumarins in response to *E. heraclei* infection by regulating key genes such *OMT, UGT*, and *PT*.

## Discussion

Dynamic metabolite variations are thought to provide a phenotypic marker of plant defense systems against biotic challenges. Plant metabolites are essential for identifying and defending against external biotic stress [28]. Plant metabolites can be classified into primary and secondary metabolites. Secondary metabolites play a crucial role in plant-pathogen interactions by directly inhibiting pathogen invasion or establishing physical barriers to prevent infection [29–31]. Combining transcriptome and metabolomic analysis offers an effective method for finding crucial metabolites and genes that respond to pathogen infections [32–34]. This study revealed significant reprogramming and redistribution of the transcriptome and metabolites following *E. heraclei* infection by RNA-seq and UPLC-MS/MS association analysis (Fig. 1a and b). A higher abundance of upregulated DEGs and DAMs was detected during the S1 and S3 infected phases (Fig. 2a and b, S1). The primary mechanism of action of *E. heraclei* infection is the up-regulation of gene expression and metabolites, similar to the transcriptome and metabolome responses of Tibetan hulless barley to powdery mildew [35]. Furthermore, the DAMs and the conserved DEGs of the three compared groups were found to be significantly enriched in the biosynthesis of various plant secondary metabolites (Figs. 4a-c and 5a-c), indicating the involvement of diverse secondary metabolites and related genes in the plant response in response to powdery mildew infection [36]. Subsequent investigations revealed that coumarin metabolites were the primary focus in accumulating these secondary metabolites (Fig. 4d).

### *E. heraclei* infection reprograms coumarin metabolism, and more coumarin metabolites accumulate in the infection site

The ortho-hydroxylation of cinnamates is the initial step in the synthesis of coumarins, an essential family of secondary metabolites and naturally occurring phytoalexin that arises from the lignin biosynthesis that is generated from the phenylpropane metabolic pathway [5]. Phenylpropanoid metabolites play crucial roles in various biological functions, mainly as signaling molecules that regulate the growth and proliferation of diverse plant pathogens [29]. The branches of phenylpropanoid metabolism produce end products such as flavonoids, hydroxycinnamic acid esters, and the precursors of lignin and lignans [37]. Several studies have focused on lignin metabolites and flavonoids that strengthen cell wall components and aid in plant resistance against powdery mildew infection [38–40]. The present study revealed that the coumarin metabolites accumulated during *E. heraclei* infection process (Fig. 4d). Simple coumarins, furanocoumarins, and pyranocoumarins are the three groups of plant coumarins according to their structural and biosynthetic characteristics. Simple coumarins have received particular attention in studies on pathogen-plant interactions [18]. However, evidence has been lacking regarding whether simple coumarins responded to powdery mildew infection.

Simple coumarins accumulate in infective areas of pathogens, often resulting in fluorescence emission [13, 41]. In the present study, the infective zones of powdery mildew showed high blue fluorescence under UV light, and both total and simple coumarins significantly accumulated in these areas (Fig. 6a-c, e). The results of this study highlighted the possibility that infection with *E. heraclei* could change the way simple coumarins accumulate up and distribute.” Simple coumarins are well known to come from the glycolytic pathway, and it is thought that an *E. heraclei* infection upsets the source-sink balance by competing with carbohydrates produced during photosynthesis and, therefore, affecting coumarin production [42]. The accumulation patterns of 113 coumarin metabolites further supported this hypothesis, demonstrating a dramatic response of simple coumarins to *E. heraclei*, with significant accumulation in S1 and S3 leaves (Fig. 6d-f and g). These results supported earlier research demonstrating the role of simple coumarins in plant resistance to fungal infections by indicating that these metabolites positively controlled *H. moellendorffii* resistance in response to *E. heraclei* [13, 14, 43].

In contrast, the accumulation of furanocoumarins and pyranocoumarins, including psoralen, bergaptol, decursinol, and rutarin, was significantly suppressed in the leaves of S1 and S3 (Fig. 6d-f). Although previous studies have identified furanocoumarins as defense compounds present as linear or angular isomers in Apiacae plants [26], there was no clear evidence of their response to powdery mildew infection in this study. Furanocoumarins and pyranocoumarins are derived from umbelliferone by the PT’s addition of the prenyl group [27, 44]. Previous studies in *Ruta graveolens* have reported that furanocoumarins form after adding a prenyl group to umbelliferone, detected in *R. graveolens*, whereas no scopoletin was detected [19]. Hence, it is possible that *E. heracle*i infection disrupted the production of furanocoumarins and pyranocoumarins by affecting the enzymatic process of umbelliferone, resulting in a higher conversion of umbelliferone into alternative coumarins, including esculetin, scopoletin, and scopoline, as a defensive reaction to combat the *E. heraclei* infection. The main novel findings of this work indicate that coumarins obtained from *H. moellendorffii* can potentially inhibit the propagation of powdery mildew infection. To our current understanding, powdery mildew can only be controlled by osthol, a natural coumarin compound derived from the dried fruits of *Cnidium monnieri* (L.) Cusson [3]. The functional significance of the identified simple coumarins on the efficacy of the powdery mildew was higher in coumarin-rich *H. moellendorffii*. Therefore, umbelliferone, esculetin, and scopoletin are potential natural fungicides promising in artificial cultivation. These substances could be used as alternatives to synthetic fungicides to control powdery mildew.

### HmF6’H1 exhibits ortho-hydroxylation activity and is in response to *E. heraclei* infection

The biosynthesis of simple coumarins involves the participation of several catalytic enzymes, including *4CL*, *F6’H*, and *UGT* (Fig. 7a) [14, 20, 45]. Potential candidate *4CL* (transcript_14261 and 10,803), *UGT* (transcript_17572 and 18,505), and *F6’H* genes (transcript_20417, 22,292, and 21,346) were identified that were strongly induced following *E. heraclei* infection (Fig. 7b and c). The 4CL is one of the critical branch point enzymes in the phenylpropanoid pathway. It has been characterized by various plants for their role in biotic stresses by regulating the biosynthesis of lignins, flavonoids, and other compounds [46]. In the study, it can be assumed that the 4CL enzymes may regulate phenylpropanoid biosynthesis to participate in *E. heraclei* infection, while specific metabolites need further study. The UGT genes could catalytic the biosynthesis of cellulose and callose at the plasma membrane to cope with biotic stresses [47]. It was shown that the Arabidopsis CALS12/GSL5 gene is responsible for producing papillary and wound-induced callose in response to powdery mildew stress [48, 49]. Additional data is required for the study to investigate if the *UGT* genes can catalyze scopoline production as a response to *E. heraclei* infection. The F6’H enzyme is widely acknowledged as a pivotal gene manufacturing simple coumarins via ortho-hydroxylation. Its role has been extensively studied in different plant species [18]. The *HmF6’H1* gene was successfully cloned in this study, and its functions were explored. Hydroxylase is commonly regarded as a soluble protein distributed in the cytosol, similar to the upstream 4CL protein and downstream coumarin synthase protein [50, 51]. A subcellular localization investigation was performed for HmF6’H1 to validate this hypothesis, which yielded results consistent with those obtained for the *CtF6’H* gene in *Clematis terniflora* DC (Fig. 8d) [52]. Previous studies have shown that the F6’H proteins exhibit strict substrate preferences. In this study, HmF6’H1 protein catalyzed the ortho-hydroxylation of p-coumaroyl-CoA and caffeoyl-CoA, forming umbelliferone and esculetin, respectively (Fig. 8f). The results deviated from previous reports indicating that F6’H proteins exhibit ortho-hydroxylation activity toward feruloyl-CoA to produce scopoletin [5, 13, 19, 52]. The variation in key residues within the structural domain may explain this discrepancy as it affects the specificity of the substrate (Fig. 8a-b). The catalytic mechanism and substrate specificity of enzymes depend on the structural characteristics, particularly the variation in residues 179 to 182 of AtF6’H1 compared to residues 174 to 177 of RgC2’H. These differences lead to changes in the active site pocket and the ability to select substrates [53]. Further molecular analysis of ortho-hydroxylases will provide additional insights into the evolutionary aspects of *H. moellendorffii* coumarins.

### HmF6’H1 positively regulated *H. moellendorffii*resistance to *E. heraclei* by regulating simple coumarins biosynthesis in infected leaves

The defense mechanism of the *F6’H* genes against pathogen invasion is intricate. The *F6’H* genes regulate the accumulation of simple coumarin to fight pathogen attacks. The specific types of simple coumarins produced in response to pathogens appear to rely on the parasitic nature of the pathogen [13, 20, 54]. For example, infection by the necrotizing fungus *Fusarium* results in the up-regulation of umbelliferone and its β-glucoside, skimmin, with concomitant induction of the *Ib2* gene in sweet potato [20]. However, *AtF6’H1* in *Arabidopsis thaliana* regulates scopoletin levels to defend against *Hyaloperonospora arabidopsidis*, a biotrophic downy mildew pathogen [54]. In the catalytic process, plant hormones, acting as key signaling molecules, are essential for regulating F6’H gene expression [13, 54, 55]. It is widely recognized that every hormone triggers a distinct molecular pathway, which is determined by the type of pathogen parasitism [56]. This may explain the differential accumulation of simple coumarins catalyzed by *F6’H* genes in response to pathogen infection.

**Fig. 10:**
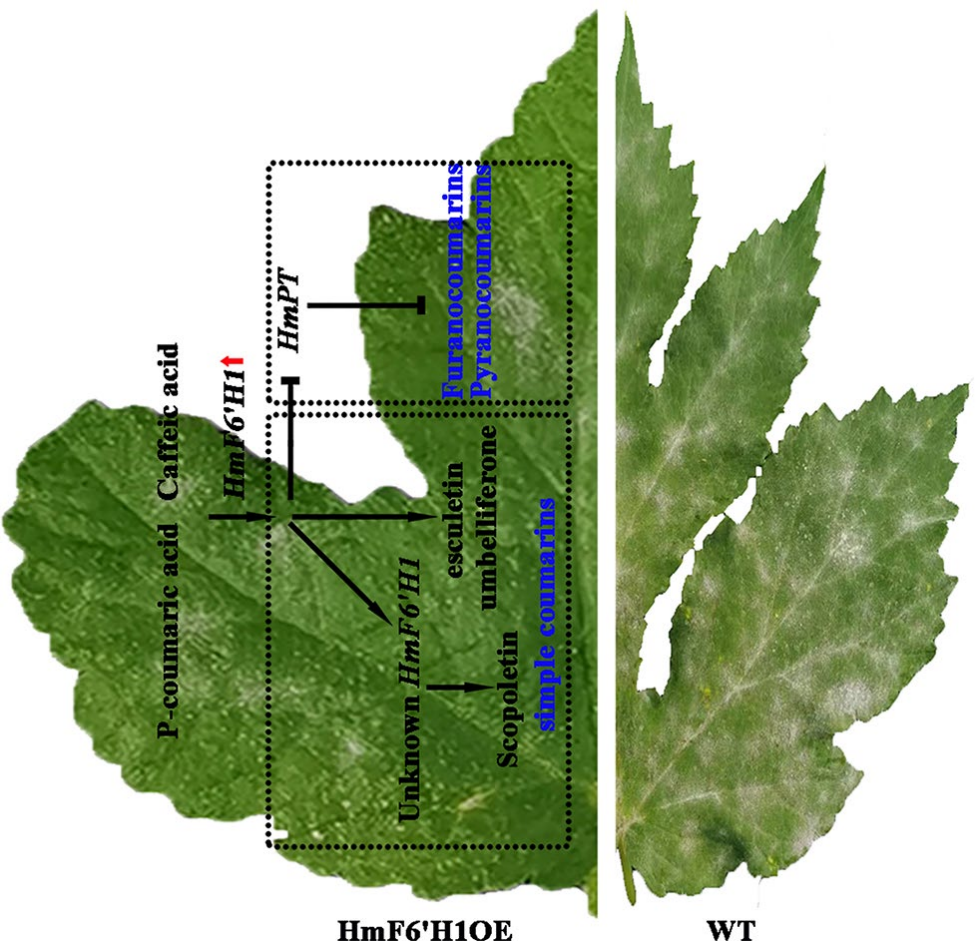
Working model of regulating HmF6’H1**-** induced simple coumarins production after *E. heraclei* infection. The HmF6’H1 proteins demonstrated ortho-hydroxylation capability towards p-coumaroyl-CoA and caffeoyl-CoA, forming the anticipated products, umbelliferone and esculetin. After *E. heraclei* infection, the over-expression of the *HmF6’H1* gene inhibited the biosynthesis of furanocoumarins and pyranocoumarins by suppressing PT gene expression, resulting in increased levels of simple coumarins, including umbelliferone, esculetin, and scopoletin, to counteract *E. heraclei* infection

Gene transient expression technology is a crucial technique used to investigate the function of plant genes. It involves transient expression vectors introducing foreign genes into host cells to achieve rapid and efficient expression or suppression of the target genes. Nevertheless, the unattached external DNA does not become part of the host cell’s chromosomal DNA, thereby preventing stable inheritance [57]. Horticultural plant leaves, fruits, and roots are also gradually used to study transient expression systems [58–60]. In this study, overexpressing HmF6’H1 reduced the occurrence of powdery mildew *H. moellendorffii* (Fig. 9a-b and e-f). The levels of umbelliferone, esculetin, scopoletin, and scopolin were significantly higher in infected HmF6’H1OE plants compared with infected WT plants (Fig. 9c). According to the findings, HmF6’H1 functioned as a resistance gene to help *H. moellendorffii* combat infection caused by *E. heraclei*. Interestingly, scopoletin content was higher in infected HmF6’H1OE than in infected WT and uninfected HmF6’H1OE plants (Fig. 9c). Based on the catalytic activity of HmF6’H1, it was hypothesized that overexpression of HmF6’H1 after *E. heraclei* infection might enhance the expression of essential genes like *OMT* and *UGT*. This, in turn, would result in the synthesis of more scopoletin, with umbelliferone and esculetin acting as substrates for the catalytic reaction. However, the expression levels of *OMT* genes (except for *HmOMT23468*) were significantly inhibited in infected HmF6’H1OE plants (Fig. 9 g). According to the findings, the *HmOMT23468* gene may be essential to the response to powdery mildew infection among the OMT gene family. Other *OMT* gene functions to encourage scopoletin synthesis may be carried out by it. The functional function of the gene *HmOMT23468* in coping with powdery mildew infection is therefore worth investigating. Furthermore, it is impossible to overlook the possibility that *HmF6’H1* overexpression or infection with *E. heraclei* led to upregulating other *HmF6’H* genes, which might have catalytic activity towards scopoletin. *HmUGT17572* and *HmUGT13977* genes may be responsible for the higher scopolin content in infected HmF6’H1OE plants than in infected WT plants. According to earlier research, the *UGT* genes are in charge of cell wall synthesis in reaction to stress caused by powdery mildew [48, 49]. This study clarifies that the UGT genes may confer plant resistance to powdery mildew by regulating scopoline accumulation. A pronounced decrease in the content of furanocoumarins and pyranocoumarins was observed in S1 and S3 infected periods (Fig. 7a). Therefore, the expression levels of crucial rate-limiting enzyme PT genes were measured. All *PT* genes were significantly down-regulated in infected HmF6’H1OE plants while upregulated in uninfected HmF6’H1OE plants (Fig. 9i). In soybean, the GmPT01 gene conferred partial resistance against stem by catalyzing multiple forms of phytoalexin glyceollins [61], while, the research about the PT genes in responding to powdery mildew stress was lacking. The results seemed to imply that *HmPTs* may benefit from powdery mildew infection by competing umbelliferone substrate with esculetin, scopoletin, and scopoline. The results suggested that by affecting the catalyzed pathway of umbelliferone after *E. heraclei* infection, *HmF6’H1* overexpression further aggravated the disruption of furanocoumarin and pyranocoumarin production. This theory corresponds with earlier results in *Ruta graveolens* [19].Therefore, the *HmF6’H1* gene has been carried out as a resistance gene in *H. moellendorffii* in reaction to an infection by *E. heraclei*. Umbelliferone, esculetin, and scopoletin are among the simple coumarins that accumulate to combat *E. heraclei* infection when the *HmF6’H1* gene is overexpressed since it suppresses the production of furanocoumarins and pyranocoumarins by reducing *PT* gene expression. Other *HmF6’H* genes may be involved in the process (Fig. 10). The study provides a new understanding of the efficacy of feruloyl-CoA 6 ' -hydroxylase on the mode of action for powdery mildew and provides a valuable candidate gene for *H. moellendorffii* breeding of resistance to powdery mildew.

Mining the *HmF6’H* gene and validating its function is essential for preventing and controlling powdery mildew in coumarin-rich crops. The HmF6’H1 gene cloned in this study will be a valuable resource for future studies of powdery mildew resistance and coumarin-rich crop breeding programs. In the meanwhile, it has been shown that simple coumarins have an anti-powdery mildew effect, which offers a desirable natural ingredient for the development of botanical fungicides and directs the production of green prevention and control of powdery mildew in coumarin-rich plants.

## Conclusions

The transcriptomic and metabolic responses of *H. moellendorffii* leaves infected with *E. heraclei* were investigated. Subsequent investigation revealed that the accumulation of these secondary compounds mostly revolved around coumarin metabolites. Identifying *HmF6’H1*, a Feruloyl CoA 6’-hydroxylase pivotal in the biosynthesis of the coumarin basic skeleton through ortho-hydroxylation, was a significant outcome. Increased expression of the *HmF6’H1* gene led to higher levels of simple coumarins, which inhibited the production of furanocoumarins and pyranocoumarins by decreasing *PT* gene expression. This enhanced the resistance of *H. moellendorffii* to powdery mildew. The results have confirmed that the *HmF6’H1* gene is crucial in helping *H. moellendorffii* combat *E. heraclei* infection. This provides additional proof of the involvement of feruloyl-CoA 6’-hydroxylase in the catalysis of different forms of simple coumarins.

## Materials and methods

### Plant material

Highly susceptible three-year-old *H. moellendorffii* plants were selected from the germplasm garden located in Haerbin City, China (Fig. 1a). The plants were cultivated in the greenhouse at the Gardening Test Station (Haerbin City, China). They were exposed to a 16-h light and 8-h dark photoperiod, and the temperature ranged from 25℃ during the day to 18℃ at night. The plants were planted on May 8, 2022. The powdery mildew isolate, identified as *E. heraclei* [2], was isolated and cultivated from infected *H. moellendorffii* leaves.

### UPLC-MS/MS metabolite analysis of *H. Moellendorffii* leaves infected by *E. heraclei*

Susceptible plants were infected with the powdery mildew using two spore suspension concentrations (1 × 10^6^ and 3 × 10^6^ conidia/mL, counted using a hemocytometer). They were cultivated with a 16/8 h (light/dark) photoperiod and a temperature range of 25/18℃ (day/night) in the light incubator (BSG-400, Boxun). Leaves infected with *E. heraclei* showing different levels of disease were collected. In the first stage (S1), the powdery mildew colonies covered less than 10% of the entire leaf surface. In the third stage (S3), the powdery mildew colonies covered 30–50% of the leaf area. The control group (Fig. 1b) consisted of leaves that were treated with water (S0) and collected. All leaf samples were collected at the same time point. Collected leaf samples were freeze-dried in a freeze-dryer (Scientz-100 F, SCIENTZ, China) and ground to a powder using the grinder (MM 400, Retsch, Germany). A total of 50 mg of the powder was extracted with 1.2 ml of 70% pre-cooled methanol internal standard extraction solution. Following vortexing and centrifugation, the liquid above the sediment was eliminated, passed through a filter with a pore size of 0.22 μm, and quantified using UPLC-MS/MS with three biological replicates. Metabolic heatmaps and association analysis of sugar metabolites were conducted using Metware Cloud, a freely accessible online platform for data analysis (https://cloud.metware.cn).

### Transcriptome sequencing of *E. heracleid*-infected *H. moellendorffii*

Various stages of disease progression (S0, S1, and S3) in *H. moellendorffii* leaves were collected for RNA-seq analysis on the Illumina HiSeq™ 2500/MiSeq (Illumina, USA) with three biological replicates (Fig. 1b). Furthermore, various *E. herclei*-infected *H. moellendorffii* samples, such as roots, stems, leaves, flowers, and seeds, were gathered for SMART sequencing using the PacBio platform. Subsequently, a quantity of approximately 50 mg of frozen tissue was utilized for the extraction of total RNA using an OmniPlant RNA Kit (CWBIO, China), following the instructions provided. In the RNA-seq analysis, the NEBNext, Oligo (dT)25 beads from NEB, USA were employed to selectively enhance the presence of mRNA in a 50ul total RNA sample. Subsequently, a NEBNext, UltraRNA Library Prep Kit (Biolabs, China) for Illumina from NEB was utilized to create an mRNA library from these enriched samples, following the provided instructions. Use the SMARTer PCR cDNA Synthesis Kit (Takara Bio, Japan) to reverse transcribe mRNA to cDNA, PCR to amplify the enriched synthesized cDNA, and optimize by cycling to determine the best conditions for PCR. The raw data quality was evaluated using the FASTQC v.0.11.8 program (https://sourceforge.net/projects/fastqc.mirror/files/v0.11.8/). Illumina adapters and bases with quality scores < 20 were trained using Cutadapt [62, 63]. The resulting clean reads were mapped to clean transcripts to correct consensus transcripts. After removing redundant sequences with CD-HIT software [64], the unigenes were further analyzed using the Non-Redundant Protein Sequence Database (Nr), Swis-Prot, Protein family (Pfam), Gene Ontology (GO) and Kyoto Encyclopedia of Genes and Genomes (KEGG) databases for functional annotation. The unigenes served as reference sequences, and the clean reads were aligned for further assessment of gene expression. Afterward, DEseq2 was utilized to perform differential expression analysis, with the criteria of |log2Fold Change| ≥ 1 and FDR < 0.05, to identify differentially expressed genes (DEGs) [65].

The K-means and Mfuzz packages were initially developed as clustering methods for analyzing gene expression or protein expression profile data. The technique enables the grouping of transcriptome and metabolome data according to their expression patterns. This allows for studying how genes and metabolites change over time or in various tissues, providing insights into their possible connections [66, 67]. The expression profiles of the *H. moellendorffii* DAMs and DEGs were performed using the k-means and Mfuzz methods. The present study separately standardized the data (z-score) for DEGs fpkm values and logarithmic processing (log10) for DAMs relative content. All samples were divided into 3 groups (S0, S1, and S3). The k-means were performed using the Metware Cloud (https://cloud.metware.cn), and the process calculates the optimal number of clusters according to the Calinski-Harabasz algorithm.

### Identification of crucial candidate genes involved in simple coumarin metabolism

The 4CL, F6’H, and UDPGT proteins were screened based on the Pfam and GO databases. Subsequent heatmaps and clustering analyses of these genes were performed using Metware Cloud (https://cloud.metware.cn). Furthermore, a regulatory network analysis was performed by combining Pearson correlation (r^2^ ≥ 0.95, p-value ≤ 0.05) between crucial candidate genes and simple coumarins using the Metware Cloud. The above analysis was performed using the fpkm values of all identified 4CL, F6’H, and UDPGT genes and the relative concentrations of umbelliferone, esculetin, scopoletin, and scopoline. Cytoscape software was used to visualize complex networks.

### Subcellular localization

The full-length coding sequences of *HmF6’H1* (excluding the stop codon) were individually cloned into the 35 S-sGFP vector. These recombinant plasmids were then transiently expressed in *Nicotiana benthamiana* via *Agrobacterium*-mediated transformation using the GV3101 strain (WeiDi, Shanghai). The resulting GFP fusion proteins were visualized using a laser-scanning confocal microscope (FV3000, Olympus, Japan).

### Production of coumarins in *E. coli* Rosetta-gami B

The complete coding sequences of *HmF6’H1* and *Os4CL* (*Oryza sativa* genes) were separately cloned into pGEX-4T-1 vectors. Each construct was transformed into *E. coli* Rosetta-gami B (WeiDi, Shanghai). The resulting strains were cultured in LB liquid medium with 50ug/mL ampicillin for 16 h at 37℃. Then, the cultures were inoculated into fresh LB with 50ug/mL ampicillin, reaching an OD600 of 1.2, and mixed in equal volumes. The induction method, using isopropyl β-D-1-thiogalactopyranoside (IPTG), was carried out as previously described [68]. At a 400 µM concentration, the substrate (p-coumaric acid, caffeic acid, or ferulic acid) was added. The resultant culture was shaken and incubated for 12 h at 30 °C. Following centrifugation, the suspension was mixed with an equivalent volume of ethylacetate and shaken for 12 h at 30℃. Afterward, 1 ml of methanol was used to dissolve the residual residue after the recovered organic phase evaporated to dryness.

The standards of umbelliferone, esculetin, scopoletin, p-coumaric acid, caffeic acid, and ferulic acid were obtained from Sigma (St. Louis, MO, America). Samples were analyzed using ultra-high-performance liquid chromatography-quadrupole time-of-flight mass spectrometry (UPLC I-Class/Xevo G2-XS QTOF, Waters, America). The Acquity uplc®beh C18 column was used with water as the mobile phase A and acetonitrile mobile phase B. The column temperature was maintained at 40℃, and the flow rate was 0.3 mL/min. The conditions of the mass spectrometry included electrospray ionization (ESI), positive ion scanning mode, a capillary voltage of 2.5 kV, a sample cone pore voltage of 40 V, an ion source default voltage of 80 V, a temperature of 120 °C for the ion source and 350 °C for the desolventization, a flow rate of 50 L/h for the cone pore gas, an 800 L/h flow rate for the desolventization gas, and a m/z range for the mass scanning.

### Transient transformation of *H. moellendorffii*

The GV3101 *Agrobacterium* strain (WeiDi, Shanghai) was used to convert the recombinant plasmids. Afterward, the cells were collected and diluted to an optical density of OD600 of 0.5. The suspended cells were cultured in the dark for 3 h and transformed into *H. moellendorffii* leaves using the vacuum infiltration method. After two days of culture at 22℃, *H. moellendorffii* leaves were inoculated with the *E. heraclei* spore suspension at a concentration of 1 × 10^6^. The plants were then cultivated in the light incubator with a 16/8 h (light/dark) photoperiod and a temperature range of 25/18℃ (day/night). After injection, samples were obtained to evaluate the levels of coumarin and gene expression. Finally, the disease condition of *E. heraclei* was assessed using ocular observation.

### Coumarin content measurement using high-performance liquid chromatography (HPLC)

The coumarin extraction method was performed as previously described [19]. Analytical testing was conducted on the samples using a Thermo Ultimate 3000 HPLC system manufactured by Thermo Fisher Scientific (USA). The HPLC system was equipped with a photodiode array (PDA) detector and a Varian C18 reversed-phase column. The samples were injected using a 20 uL injection volume. The mobile phases comprised acetonitrile and a 0.05% phosphoric acid solution in a 25:75 ratio. The column temperature was maintained at 30 °C, and the flow rate was set at 1 mL/min. Detection and separation were monitored at 320 nm.

### E. heraclei infection in H. moellendorffii

Disease classes are based on the occurrence of powdery mildew in H. moellendorffii and reference to the powdery mildew classification of wheat crops [69]. Disease index = ∑(disease scale × disease leaves number)/(total leaves number × maximum disease scale). The average number of conidiophores per colony was used to indicate the degree of susceptibility [70]. About 5 well-isolated colonies from infected *H. moellendorffii* leaves were randomly chosen for calculating the number of conidiophores under the inverted microscope (DMiL, Leica, Germany). Trypan blue staining was used to observe the powdery mildew infection. For each plant, 2 to 4 0.5 cm by 1 cm leaves were cut, stained, and heated for 10 min using 0.067 mg/ml Tepan blue staining solution. After the plants cooled naturally, 2–3 ml chloral hydrate was added for decolorization and microscopic observation was done using an inverted microscope (DMiL, Leica, Germany) [71].

### Quantitative real-time PCR (qRT-PCR) analysis

TransZol UP (TransGen Biotech, China) was utilized to extract plant RNA. Following this, cDNA Synthesis SuperMix (TransGen Biotech, China) was used to produce the cDNA. The reference gene for normalization was *hmActin*. qRT-PCR assays were performed using a Bio-Rad Thermal Cycler (qTOWER3G. Analytikjena, Germany). The relative gene expression was calculated using the 2^−△△CT^ method.

### Statistical analysis

All data were presented as the means ± standard deviation (SD) of three or more biological replicates. One-way analysis of variance (ANOVA) or Student’s t-test was used to evaluate the significant differences, with a significance level of *p* < 0.05.

### Electronic supplementary material

Below is the link to the electronic supplementary material.

Supplementary Material 1

Supplementary Material 2

## Data Availability

The raw RNA-seq reads generated by this study are publicly available at the NCBI Short Read Archive (SRA) and BioProject accession PRJNA1032719 (https://www.ncbi.nlm.nih.gov/Traces/study/?acc=PRJNA1032719). Other data supporting the findings of the study are available within its supplementary materials.
