# Supplementary figures and images for: Combined metabolome and transcriptome reveal HmF6’H1 regulating simple coumarin accumulation against powdery mildew infection in Heracleum moellendorffii Hance
Source: BMC Plant Biol. 2024 Jun 6;24:507. doi: 10.1186/s12870-024-05185-3 (PMC11155083; doi:10.1186/s12870-024-05185-3)

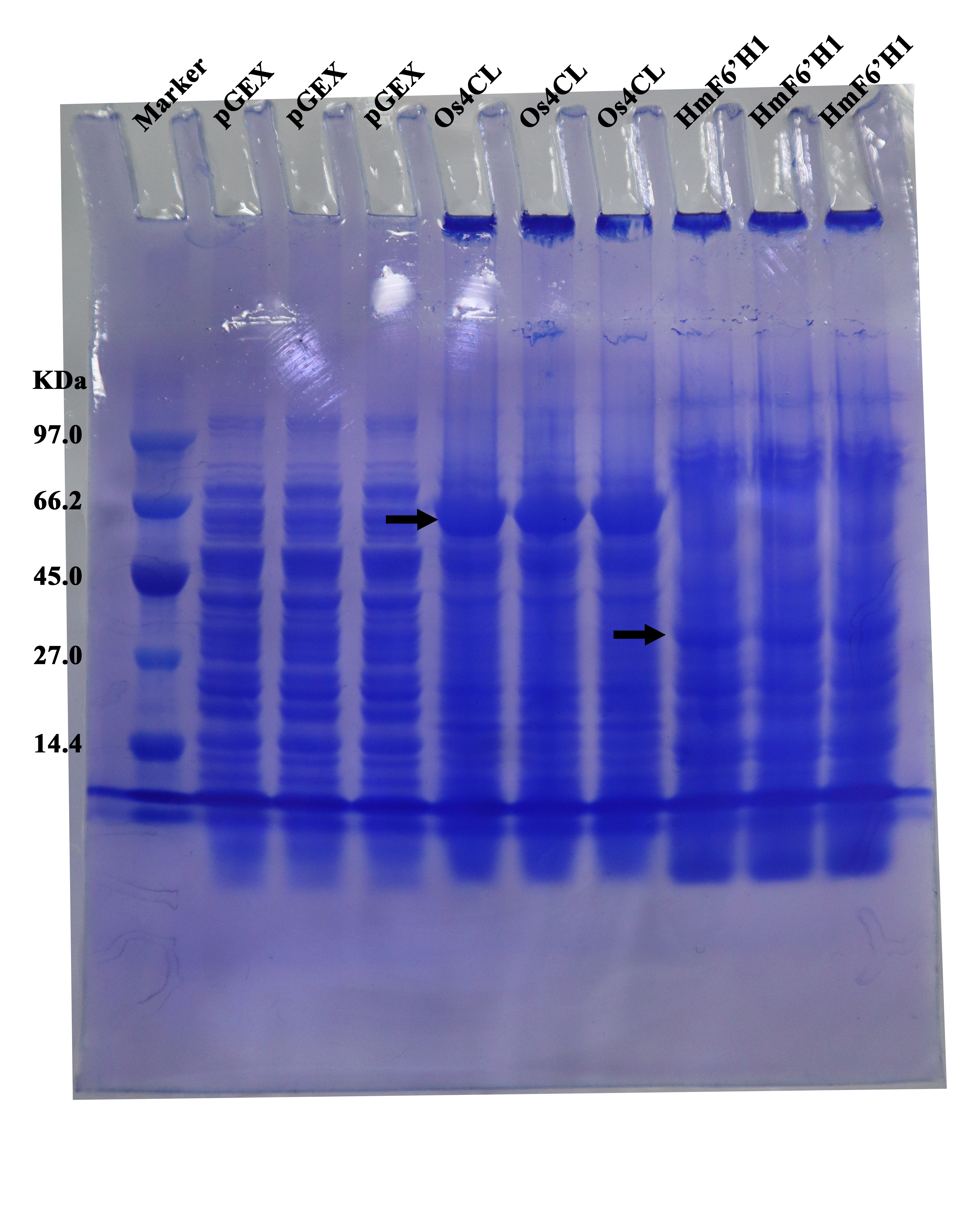

Supplement: Supplementary file 2 — Supplementary Material 2 [file 12870_2024_5185_MOESM2_ESM.jpg]
